# Profile and outcome of patients with emergency complications of renal failure presenting to an urban emergency department of a tertiary hospital in Tanzania

**DOI:** 10.1186/s12873-019-0229-2

**Published:** 2019-01-22

**Authors:** Erasto Sylvanus, Hendry R. Sawe, Biita Muhanuzi, Elly Mulesi, Juma A. Mfinanga, Ellen J. Weber, Said Kilindimo

**Affiliations:** 1 Emergency Medicine Department, Muhimbili University of Health and Allied Science, P.O Box 65001, Dar es Salaam, Tanzania; 2 Emergency Medicine Department, Muhimbili National Hospital, Dar es Salaam, Tanzania; 3 Department of Emergency Medicine, University of California, San Francisco, San Francisco, CA USA

**Keywords:** Renal failure, Emergency complications, Dialysis, Emergency department

## Abstract

**Background:**

Renal failure carries high mortality even in high-resource countries. Little attention has been paid to renal failure patients presenting acutely in emergency care settings in low-to-middle income countries (LMIC). Our aim was to describe the profile, management strategies and outcome of renal failure patients presenting with indications for emergent dialysis to an urban Emergency Department (ED) in a tertiary public hospital in Tanzania.

**Methods:**

This was a prospective cohort study of consecutive patients (age ≥ 15 yrs) presenting to the Emergency Medicine Department of Muhimbili National Hospital from September 2017 to February 2018. All patients with renal failure and complications requiring acute dialysis were included. A structured data collection sheet was used to gather demographics, clinical presentation, management strategies and outcomes. Data were summarized with descriptive statistics. Logistic regressions were performed to determine factors associated with receiving dialysis and with mortality.

**Results:**

We enrolled 146 patients, median age was 49 years (IQR 32–66 years), and 110 (75.3%) were male. Shortness of breath 67 (45.9%) and reduced urine output 58 (39.7%) were the most common presenting complaints. The most common complications were hyperkalemia 77 (53%), uremic encephalopathy 66 (45%) and pulmonary edema 54 (37%). All patients were hospitalized, and 61 (42%) received dialysis. Overall mortality was 39% (57 patients); the mortality in non-dialysed patients was 53% vs. 20% (*p* < 0.0005) in those receiving dialysis. 54% of patients with health insurance were dialyzed, compared to 39% who paid out of pocket (adjusted OR = 0.3, 95%CI: 0.1–0.9). Patients (≥55 years) were less likely to be dialysed (adjusted OR = 0.2 [0.1–0.9]). Independent predictors of mortality were vomiting (OR = 6.2, 95%CI: 1.8–22.2), oliguria (OR = 3.4, 95%CI: 1.2–9.5), pulmonary edema (OR = 4.6, 95%CI: 1.6–14.3), creatinine level > 1200umol/L (OR = 5.0 95%CI: 1.4–18.2), and not receiving dialysis (OR = 8.0, CI: 2.7–23.5). Female sex had a lower risk of dying (OR = 0.13, CI: 0.03–0.5).

**Conclusions:**

In this ED in LIC, acute complications of renal failure created a need for ED stabilization and emergent dialysis. Overall in-hospital mortality was high; significantly higher in undialysed patients. Future studies in LICs should focus on identification of categories of patients that will do well with conservative therapy.

## Background

Renal disease is common worldwide. Chronic Kidney Disease (CKD) affects up to 16% of the world population [1], while Acute Kidney Injury (AKI) is associated with 5–20% of hospital admissions [2, 3]. Renal failure carries a high mortality rate in both high and low resource countries [4]. Emergency departments are frequently the first site of care for patients who develop acute kidney injury and those who develop complications from chronic kidney disease. Appropriate management of these patients can be life-saving.

In developing countries, the prevalence of CKD is similar to that of high resource countries, 13·9%, while that of AKI is up to 7% [3]. To a certain extent the etiologies are similar to those in high resource countries with non-communicable diseases such as hypertension, diabetes, lipid disorders being major contributors. However, in LMIC’s, communicable diseases such as kidney infections, schistosomiasis, leishmaniasis and Human Immunodeficiency Virus (HIV) infections are also significant risk factors for the development of renal injury [5]. Poor access to care for some of these diseases (e.g. hypertension) result in earlier onset of CKD than in high resource countries and late presentation and financial inability to access advanced care such as dialysis and renal transplant contribute to higher mortality rates from renal disease [4].

Management of acute complications of renal failure at the emergency medicine department (EMD) rests on early recognition, early resuscitation, and treatment of the underlying causes to prevent further renal damage [5]. Patients present with life-threatening electrolyte imbalance, commonly hyperkalemia, severe metabolic acidosis and uremic complications such as pulmonary edema, uremic pericarditis and encephalopathy. Non-invasive ventilation such as continuous positive airway pressure or intubation may be needed to manage patients with respiratory failure due to fluid overload. However, these are often temporizing measures, and dialysis is often necessary.

In Tanzania, as in much of sub-Saharan Africa, the infrastructure for emergency care is still in development. Dialysis availability is limited throughout the country [6, 7]. Recent studies on challenges and outcomes of hemodialysis showed a substantial challenges in accessing dialysis services, which is compounded with the high cost related to its utilization [8]. Muhimbili National Hospital (MNH), a tertiary referral hospital, is among few public hospitals in the country with full capacity emergency medicine department (EMD) staffed by Emergency Medicine specialists, which is in the position to provide stabilization for patients with acute complications of renal failure. However, little is known about the number and characteristics of patients presenting to the EMD, how they are managed or the outcome of EMD treatment. Moreover, it is unknown how many who meet requirements for dialysis receive it, and how many patients survive to discharge. Determining the numbers of patients presenting with acute complications of renal failure, their clinical characteristics and requirements for dialysis, and their current outcomes can help improve management and direct scarce resources appropriately.

## Methods

### Study design

This was a prospective cohort study of patients aged 15 years and above presenting to the EMD of the national referral hospital in Tanzania from September 2017 to February 2018, with acute symptoms of renal failure requiring emergent dialysis.

### Study setting

The study was conducted at the EMD and nephrology unit of Muhimbili National Hospital (MNH), which is a tertiary public hospital located in Dar es salaam, Tanzania. This hospital has approximately 1500 beds; patients are referred from different districts and regional facilities across Tanzania. At the EMD-MNH, more than 1200 patients attend weekly. The dialysis unit at MNH has about 40 machines running about 80 dialysis sessions per day. The hospital offer emergency dialysis to all patients with emergency presentations of renal failure (including symptomatic hyperkalemia, pulmonary oedema, uremic encephalopathy and pericarditis), patients with chronic renal failure, receives care in line with existing national guideline. All patients with emergency conditions receives care regardless of the ability to pay, while regular patients receives care as per the cost sharing (Government subsidized cost), insurance, exemption and private categories.

### Participants

We defined renal failure using RIFLE criteria [9]. Since RIFLE criteria use creatinine levels and urine output measured at 12 or 24 h intervals, and this was not feasible in the emergency setting where a patient might spend less time, for this study we used reported or observed oliguria or anuria for 12 h and above. Eligible participants were those with renal failure who presented to the ED with one or more acute complications such as fluid overload, severe electrolyte imbalance, acute drug intoxication, severe metabolic acidosis and uremia that required emergent dialysis. Patients with pregnancy, trauma, obstructive oliguria and those who did not consent were excluded.

### Study protocol

A researcher was scheduled for 12 h a day throughout the study period; shifts alternated days and nights. The research also collected information of all patients by chart reviewing and identified patients that came in during the off hours**.** During these periods, all patients presenting to the resuscitation area of the ED were screened by real-time chart review for provisional and final diagnoses and rounding with physicians; the diagnosis of renal failure was confirmed by having elevated serum creatinine levels. We then determined which patients had complications requiring dialysis and these patients were enrolled.

After obtaining patients consent, a structured data collection sheet was used to gather demographic information, clinical presentation, management strategies and outcomes. Laboratory results were followed up through electronic system to get the data for creatinine levels in case the results were not recorded in the electronic medical record system at ED or in the patient’s file. Patients were followed until hospital discharge or up to 30 days in the wards for the outcomes.

### Outcomes

Primary outcome was mortality rate (ED mortality and 30-days in-hospital mortality rates). Secondary outcomes were risk factors for mortality, whether or not patients received dialysis, and predictors of receiving dialysis.

### Data analysis

As there were no similar studies on the outcomes of patients admitted with complications of renal failure, we based the sample size estimate on a mortality rate of 34% for patients with AKI hospitalised in a US study [10]. Using a 95% confidence interval, a minimum sample size of 119 was calculated.

All the data collected were entered into an electronic database (Redcap), and then exported into excel sheet and transferred to the Statistical Package for Social Sciences (SPSS), version 20 for analysis. Data were summarized with descriptive statistics including median and frequencies. Logistic regression was used to determine factors associated with mortality and receipt of dialysis. Patients who died in the ED were excluded from these analyses as they could not be evaluated for dialysis access. **A** probability value of less than 5% was considered statistically significant.

## Results

### Demographics and clinical characteristics of study participants

**Fig. 1:**
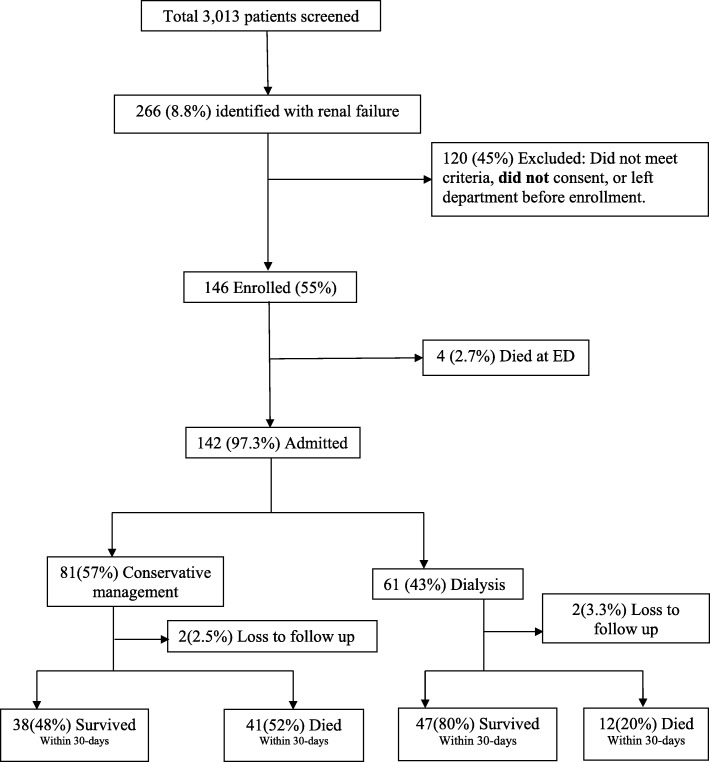
Prospective cohort flow chart of patients presenting at ED with emergency complication of renal failure

Of the 3013 patients admitted to resuscitation rooms during the study period, 266 (8.8%) had a diagnosis of renal failure (71 AKI versus 195 CKD). **(**Fig. 1**)** Among these, we enrolled 146 (55%) patients who had indications for dialysis. Majority of participants were male 110 (75.3%) and aged above 55 years 55(37.7%) while median age was 49 years (IQR 32 to 66 years). The majority 88 (62%), had not gone beyond primary school education. More than half (52%) were referred from government health facilities; 74% (108) were uninsured and paying out of pocket (Table 1). The most common complaints of participants were shortness of breath 67(45.9%), reduced urine output 58 (39.7%) and generalized swelling 51(34.9%). The main presenting complications diagnosed among study participants were hyperkalemia 77(53%), uremic encephalopathy 66 (45%) and pulmonary edema 54 (37%) (Table 2).

**Table 1:** Demographic characteristics of participants

Demographic characteristics	Number (*N* = 146)	%
Age		
15–34	36	24.6
V35–54	55	37.7
55+	55	37.7
Sex		
Male	110	75.3
Female	36	24.7
Education *		
Higher education	17	12.0
Secondary	27	19.0
Primary	88	62.0
Informal Education	10	7.0
Payment		
Insurance	38	26.0
Pay from pocket	108	74.0
Referred from **		
Private	23	15.9
Government	76	52.4
Self-referral	46	31.7
Co-morbidities		
Hypertension	66	52.0
No Known comorbidities	28	22.0
Diabetes Mellitus	25	19.7
HIV infection	12	9.4
Malignancy	4	3.1

* 4 Missing; **1 missing

Education levels were not recorded for 4 participants, referral type not recorded for 1 participant

**Table 2:** Presenting Complications of participants

Presenting Complications	*N* = 146	**%**
Hyperkalemia (K^+^ > 5.5)	77	53.0
Uremic encephalopathy	66	45.0
Pulmonary edema	54	37.0
Metabolic acidosis (PH ≤ 7.2)	28	19.0

Participants could have more than one complication. Therefore, adding the frequencies will give a larger number than *N* = 146

### Management given and outcome at ED

**Fig. 2:**
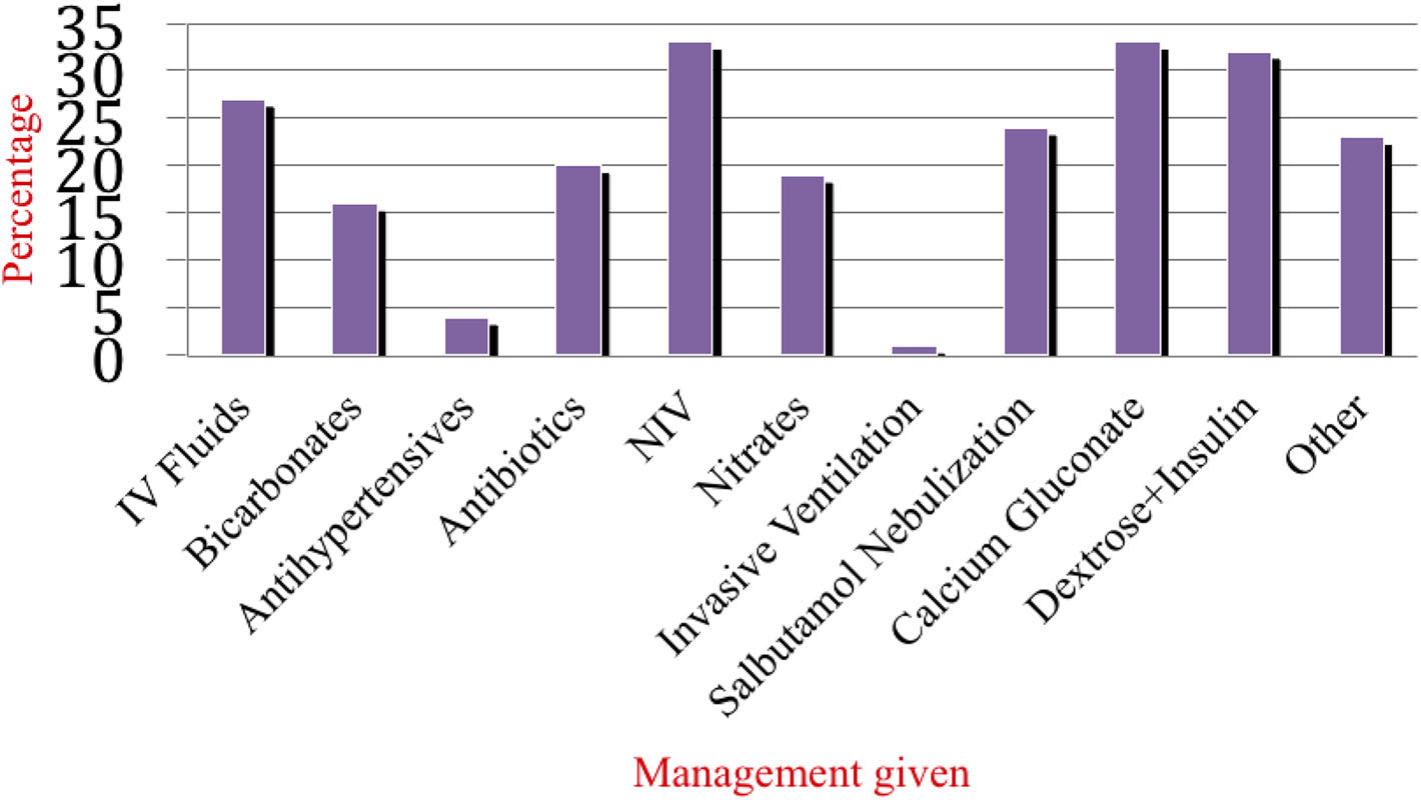
Management at EMD

Most of ED management was directed for treatment of hyperkalemia and pulmonary fluids overload **(**Fig. 2**).** The ED administered non-invasive ventilation (NIV) (33%), Calcium gluconate (33%), insulin with dextrose (32%), diuretics (30%), and nebulization with salbutamol (24%), antibiotics (20%), nitrates (19%) and sodium bicarbonates (16%). Few patients received antihypertensives (4%) and 1% of the participants received invasive ventilation and inotropes. 2.7% (4/146) patients died at ED, while 97.3% (142/146) were admitted alive.

### Frequency and predictors of dialysis

Out of 142 patients who were admitted with emergency complications of renal failure in need of dialysis, 61 (42.8%) received dialysis while 81 (57.2%) were managed conservatively.

In multivariate logistic regression analysis, lack of insurance and older age were independent predictors of not receiving dialysis (OR: 0.30; 95%CI: 0.1–0.9) and OR 0.2 95% CI 0.1–0.9), respectively (Table 3).

**Table 3:** Factors influencing access to dialysis (*n* = 142)

	Total	Dialyzed	Not Dialyzed	Multivariate Logistic Regression
	*N* = 142	*N* = 61	*N* = 81	
Age categories				
< 30	25	15 [60]	10 [40]	
30–54	63	28 [44.4]	35 [55.6]	0.7[0.2–2.3]
55+	54	18 [33.3]	36 [66.7]	0.2[0.1–0.9]
Total	142	61 [43]	81 [57]	
Mode of payment				
Insured	37	20 [54.1]	17[45.9]	
Out of pocket	105	41 [39]	64[61]	0.3[0.1–0.9]
Total	142	61 [43]	81[57]	
Creatinine (umol/L)				
< 700	32	11[34.4]	21[65.6]	
700–1200	33	13[39.4]	20[60.6]	1.3[0.3–5.4]
1201+	68	32[47.1]	36[52.9]	2.0[0.6–6.8]
Total	133	56[42.1]	77[57.9]	
Blood Urea Nitrogen (BUN) (mmol/L)				
< 20	18	9 [50]	9[50]	
20+	112	47[42]	65[58]	0.5[0.1–1.9]
Total	130	56[43.1]	74[56.9]	
Potassium (mmol/L)				
< 7	102	46[45.1]	56[54.9]	
7+	20	8[40]	12[60]	1.1[0.3–3.5]
Total	122	54[44.3]	68[55.7]	

4 participants who were lost to follow-up and 4 other patients who died at ED were not included in the analysis

### Mortality

Among all 146 enrolled patients, four patients died in the ED while 53 patients died in the hospital, giving an overall mortality rate of 39%, (95%CI 31.1–45.0%). Table 4 shows the results of univariate and multivariate analysis of risk factors for mortality. The following patient characteristics were independent predictors for mortality: vomiting at presentation (OR: 6.23; 95%CI: 1.75–22.2), oliguria (OR: 3.40; 95%CI: 1.22–9.48), body swelling (OR: 3.40; 95%CI: 1.17–9.88), altered mental status (OR: 7.48; 95%CI:1.90–29.41), pulmonary edema (OR: 4.60; 95%CI: 1.6–13.3), and serum creatinine level above 1200ummol/L (OR: 5.02; 95%CI: 1.4–18.20). Hospital management, specifically dialysis, was the strongest predictor of mortality: Among the 61 patients dialyzed, there were 12 (20.3%) deaths, and among the 85 not dialyzed, there were 45 deaths (53%). Patients who were not dialyzed were significantly more likely to die than those dialyzed. (OR: 8.02; 95%CI: 2.7–23.5) Females were less likely to die than males (OR: 0.13; 95%CI: 0.03–0.53) Table 4.

**Table 4:** Factors associated with mortality (Logistic regression analysis)

	Total	Alive	Died	Logistic Regression			
				Univariate		Adjusted	
	*N* = 142	n (%)	n (%)	OR[95%CI]	*P*-Value	OR[95%CI]	*P*-Value
Age							
< 30	26	16[61.5]	10[38.5]	1		1	
30–54	64	40[62.5]	24[37.5]	1.6[0.38–6.94]	0.932	2.81[0.44–17.89]	0.825
55+	52	29[55.8]	23[44.2]	0.7[0.17–3.24]	0.627	1.13[0.17–7.37]	0.782
Sex							
Female	34	28[32.9]	6[10.5]	0.2[0.09 0.63]	0.003	0.13[0.03–0.53]	0.004
Payment							
Insurance	37	22[25.9]	15[26.3]	1			
Pay from pocket	105	63[74.1]	42[73.7]	0.9[0.46–2.10]	0.954		
Hospital Management (*n* = 138 *)							
Dialyzed	59	47[55.3]	12[22.6]	1		1	
Not dialyzed	79	38[44.7]	41[77.4]	4.2[1.9–9.1]	0	8.02[2.7–23.5]	0
Potassium in mmol/L (*n* = 119*)							
< 7 mmol/L	98	65[66.3]	33[33.7]	1			
7 + mmol/L	21	12[82.4]	9[42.9]	1.5[0.56–3.86]	0.426		
Reduced urine output (*n* = 138*)							
Yes	57	32[37.6]	25[43.9]	1.3[0.65–2.56]	0.459	3.40[1.22–9.48]	0.005
Altered mental status (*n* = 138*)							
Yes	66	37[43.5]	29[50.9]	3.6 [1.36–9.67]	0.01	7.48[1.90–29.41]	0.004
Shortness of breath (n = 138*)							
Yes	33	20[23.5]	13[22.8]	1.3[0.69–2.63]	0.39		
Vomiting (n = 138*)							
Yes	43	29[34.1]	14[24.6]	1.6[0.71–3.42]	0.266	6.23[1.75–22.2]	0.005
Physical and Lab findings							
Pulmonary edema (n = 138*)							
Yes	54	27[31.8]	27[47.4]	1.9[0.97–3.86]	0.062	4.60[1.6–13.3]	0.005
Creatinine in umol/L (*n* = 133*)							
< 700	31	21[67.7]	10[32.3]	1		1	
700–1200	32	20[62.5]	12[37.5]	1.3[0.45–3.56]	0.663	1.52[0.40–5.75]	0.539
1201+	70	38[54.3]	32[45.7]	1.8[0.73–4.30]	0.208	5.02[1.4–18.2]	0.014
BUN Level in mmol/L (*n* = 134*)							
< 20	18	12[11]	4[25.0]				
20+	116	66[28.8]	48[42.1]	2.2[0.66–7.18]	0.199		

*N’s vary as all patients did not have all tests in the ED. 4 participants who were lost to follow-up and 4 other patients who died at ED were not included in the analysis

## Discussion

Prior studies of patients with renal failure attending emergency departments come from high-income countries (HIC), where demographics and etiology of renal failure may differ [8–13] In addition, low-to-middle income countries (LMIC) generally have poor access to primary care, and individuals often delay seeking care. LMIC have less availability of life-saving treatments, including ventilators, dialysis and IV medications. These factors can result in different management strategies and outcomes for patients. To our knowledge, there have been no previous studies in LMIC determining the number of patients presenting to the ED with emergency complications of renal failure or their characteristics.

During the study period, 266 patients presented with renal failure, approximately 8.8% of the patients attending the ED resuscitation rooms. Of these, 146 met criteria for emergency dialysis. Most patients (75.3%) in our study were males, similar to previous published studies across European countries [14, 15]. The median age was 49 years, which is higher than the average ED population, which has a median age of 30 years [16]. However this is lower than patients presenting to EDs in HIC’s [17, 18]. This could be attributed to the presence of untreated comorbidities such as hypertension, unique etiologies of renal failure in sub-Saharan Africa, and less access to preventive care.

Patients with low education levels and no health insurance made up the majority of the patients, while in HIC the majority had higher education [19]. This could be a result of the fact that MNH is a public hospital, and people with higher education and health insurance are likely to opt for other centers when seeking medical care. However, another potential reason is that those with insurance and higher education get health care more regularly, and may be on chronic dialysis, thus are less likely to present to any ED with complications of renal failure.

Similar to studies from HIC’s, the most common symptom reported was shortness of breath (45.9%) although in a US study, this was found in a higher proportion (61%) of patients [20]. There were relatively more patients with uremic encephalopathy (45%) and vomiting (24%) compared to 6.6 and 8.2% respectively in previous HIC literature [15, 20]. Most notably, more than 50% of our patients had hyperkalemia, compared to studies in HIC’s where hyperkalemia in patients presenting to ED with renal failure requiring dialysis was found in less than 10 % [19, 21]. This is likely due to the late presentation of renal failure in those not getting regular care, as well as those who have renal failure not getting dialysis.

Temporizing management given at ED suggested a significant stabilizing effect in which less than 3 % of patients died at ED while others were admitted alive to renal units to receive definitive management. This is similar to the mortality of the ED population with renal failure in HIC [22].

Our study found that, overall 30-day mortality of patients who needed dialysis was somewhat higher (39%) than in developed countries which had a mortality of around 8.8% [17]. Higher mortality found in our study can be attributed to late presentation to the hospital and lack of dialysis in more than 50% of patients who meet the indications. In developed countries more than 80 % of those admitted to renal and dialysis units receive dialysis [20].

The strongest predictor for mortality in our population was dialysis; non-dialyzed patients had more than eight times higher odds of dying than those who got dialyzed. This is consistent with a previous study done in a high income setting in which less than 10 % of those who were dialyzed died [15]. We found that mode of payment and age, were associated with the probability of being dialyzed or not. Not having health insurance (i.e. paying out of pocket), reduced the odds of dialysis by 70%. Age 55 years and above decreased the odds of dialysis by almost 80%. This is different from a study in the US which showed shortness of breath was the only factor in predicting dialysis [20, 23]. Dialysis units are limited in Tanzania and other LMIC’s and thus a form of triage occurs: it is widely accepted that more advanced age is associated with poor outcome and due to limited resources in Tanzania; the younger population is usually preferred. Unfortunately, inability to pay for dialysis is also a barrier to receiving it. Study by Meremo et al. among patients receiving dialysis at the University teaching identified potential predictors of poor outcomes to be gender, type of kidney disease, residence of patients and insurance status of patient [8].

### Limitations

This was a single-centre study, which may affect generalizability of its results. We did however reach the appropriate sample size to increase accuracy. There was also some missing patient information at medical records and uncharted documentation of point-of-care and laboratory results. Some patients may have been missed due to the fact that the research assistant was not present at the emergency department all the time, and this could underestimate the prevalence of the problem. However, as the shifts were varied in timing, we feel that this sample is representative of our population. Some screened patients were identified as potentially eligible but left the department before they could be enrolled; however, this number is small.

## Conclusion

In this ED in LIC, we found that acute complications of renal failure created a substantial need for ED stabilization and emergent dialysis. Overall in-hospital mortality was high; significantly higher in undialysed patients. Future studies in LICs should focus on identification of categories of patients that will do well with conservative therapy.

## Abbreviations

AKF: Acute renal failure
AKI: Acute Kidney Injury
BUN: Blood urea nitrogen
CKD: Chronic Kidney Disease
CPAP: Continuous positive pressure ventilation
ED: Emergency Medicine Department
EMD: Emergency Medicine Department
GFR: Glomerular Filtration Rate
INR: International Normalized Ratio
IRB: Institutional Review Board
IV: Intravenous
IVF: Intravenous fluids
LIC: Low Income Countries
